# CHDH Promotes Breast Cancer Metastasis Relying on IL17RB/CREB1 Signalling Activation

**DOI:** 10.1111/jcmm.70792

**Published:** 2025-09-10

**Authors:** Yifei Li, Yipeng Liu, Xiaowen Yang, Xinzhuang Shen, Zongjun Liu, Yuqiu Ma, Yiting Zhan, Xinyu Jiang, Chengjin Liang, Xiaoyuan Zhang, Huan Liu, Wenzhi Shen

**Affiliations:** ^1^ Shandong Provincial Precision Medicine Laboratory for Chronic Non‐Communicable Diseases, Institute of Precision Medicine Jining Medical University Jining China; ^2^ Department of Breast Surgery Dalian Women and Children's Medical Group Dalian China; ^3^ College of Clinical Medicine Jining Medical University Jining China; ^4^ Henan Key Laboratory of Immunology and Targeted Drugs, School of Laboratory Medicine Xinxiang Medical University Xinxiang China

**Keywords:** breast cancer (BC), CHDH, IL17RB/CREB, metastasis, migration

## Abstract

The involvement of Choline Dehydrogenase (CHDH) in metabolic disorders and tumour progression has garnered significant scholarly interest. However, the specific role of CHDH in the metastasis and progression of breast cancer (BC) has been less thoroughly investigated. Our research indicates that CHDH protein expression is markedly elevated in breast cancer tissues compared to normal tissues, and this expression is positively correlated with the tumour node metastasis (TNM) stage of breast cancer. Furthermore, CHDH levels were found to be significantly higher in breast cancer cell lines relative to normal breast cells. The silencing of CHDH expression resulted in a reduction of breast cancer cell migration, while the overexpression of CHDH facilitated increased migration and tumour metastasis in vivo. Investigations into the underlying mechanisms revealed that CHDH influences the expression of IL17RB and activates Cyclic‐AMP Response Element‐Binding Protein (CREB), thereby mediating breast cancer metastasis. The application of an IL17RB antibody and the CREB inhibitor 666‐15 effectively abolished CHDH‐mediated migration of breast cancer cells in vitro. These findings suggest that CHDH plays a critical role in promoting breast cancer metastasis, potentially offering new targets and strategies for the treatment of metastatic breast cancer.

**Abbreviations**
BC: breast cancer
CHDH: choline dehydrogenase
CREB: cAMP response element, CRE‐binding Protein
IL17: interleukin 17
IL17RB: interleukin 17 receptor B
MAPK: mitogen‐activated protein kinase
MSK: mitogen and stress‐activated protein kinase
PKA: protein kinase A
TNM: tumour node metastasis

## 1 Introduction

Breast cancer (BC) is one of the leading causes of cancer death in women [1]. With the development of clinical diagnosis and treatment and the increasing rate of population health screening, the treatment and prognosis of BC have steadily improved, and the 5‐year survival rate of patients has significantly increased. However, effective strategies for the treatment of advanced metastatic BC are still lacking [2]. There is an urgent need to discover new specific biomarkers or targets for the early diagnosis and targeted therapy of metastatic BC.

Human CHDH (Choline Dehydrogenase, CHDH; E.C.1.1.99.1) is a nuclear‐encoded mitochondrial enzyme that is one of the key enzymes catalysing choline metabolism on mitochondria [3]. The CHDH catalytic end product betaine is an important methyl donor [4, 5]. In terms of physiological functions, CHDH regulates free choline concentration and participates in mitochondrial autophagy [6, 7]. In addition, when choline is used as a drug, CHDH promotes the rapid metabolism of free choline, so that free choline cannot be continuously elevated and inhibit choline from exerting its drug effects [6]. In recent years, it has been found that CHDH is closely associated with the development of a variety of diseases, such as malignant tumours [8, 9], male infertility [10], dental hypoplasia [11], hyperhomocysteinemia [12] and psychiatric disorders [13]. Clinical studies have confirmed the ability of CHDH to predict response to tamoxifen monotherapy and relapse in BC patients [14]. However, the specific functional and molecular mechanisms of CHDH in BC progression are unknown.

The transcription factor cAMP Response Element, CRE‐binding Protein (CREB) belongs to the leucine zipper type of transcription factors with the potential to regulate about 4000 genes [15]. CREB1 forms homo‐ and/or heterodimers with other CREB family members or activator protein 1 to form homo‐ and/or heterodimers that mediate gene transcription [16]. Transcriptional activity has been demonstrated in a number of genes including Mitogen‐Activated Protein Kinase (MAPK) [17], Protein Kinase A (PKA) [18], among others, which phosphorylate the induction of CREB1. CREB1 has been reported to be involved in several tissue types of differentiation [19] and function as a proto‐oncogene [20, 21, 22] to promote malignant progression [23], involvement in gene fusion [24] and aberrant activation [25]. Recent studies have shown that overexpression of CREB1 can act as a transcription factor to promote the proliferation and migration of breast cancer cells [26]. However, the role of CREB1 activation in CHDH‐mediated BC progression and metastasis has not been reported.

Interleukin 17 (IL17) ligand and its family of receptors are involved in the pathogenesis of many diseases such as rheumatoid arthritis, breast cancer and psoriasis [27]. It has been shown that both IL17 and CREB are highly expressed in breast cancer and promote tumour progression [28]. Addition of IL17 to human smooth muscle cells induced CREB activation to promote cell migration and proliferation [29]. IL17 is also a major mediator of inflammation within tumours and is produced by intratumoral helper T cells 17, which promote breast cancer progression. Interleukin 17 receptor B (IL17RB) is a receptor for the pro‐inflammatory cytokines IL17B and IL17E. In the serum of breast cancer patients, IL17RB can be detected by standard ELISA assays [27]. However, whether IL17RB is involved in CHDH‐mediated BC progression is unknown.

In the present study, we investigated CHDH expression in BC. We explored the effects of CHDH knockdown and overexpression on BC cell migration in vitro and metastasis in vivo. We also performed a detailed analysis of the potential mechanisms by which CHDH affects BC metastasis. In addition, we validated the CHDH‐mediated signalling pathway using an inhibitor or specific antibody in vitro. Our study identified novel markers of BC metastasis and identified new targets and strategies for the treatment of clinical BC metastasis.

## 2 Materials and Methods

### 2.1 Online Database Analysis

The ONCOMINE online database was used to analyse CHDH expression in normal breast tissue and breast cancer tissue. GeneMANIA online database was used to analyse the set of genes related to CHDH function.

### 2.2 Human Breast Cancer Tissues

We obtained part of the breast cancer tissues and paracancerous tissues from patients who underwent surgery at Affiliated Hospital of Jining Medical University in 2021–2022. None of the patients received preoperative chemotherapy (pathological details are listed in Table S1). The specimens were placed in liquid nitrogen immediately after collection and then stored in a −80°C refrigerator. This study was reviewed and approved by the Ethics Committee of Jining Medical University (JNMC‐2021‐YX‐017).

### 2.3 Vector Construction

A CHDH‐specific short hairpin RNA (shRNA) oligo was designed and synthesised in Table S2. The shRNA was ligated into the pLV‐H1‐EF1α‐Puro vector (Cat. #B19, Biosettia, San Diego, CA, USA) to obtain the final pLV‐H1‐shCHDH‐EF1α‐Puro vector [30]. The CHDH overexpression vector pLVML‐3 × Flag‐CHDH‐Puro was obtained from Fenghui Biology (Changsha, China) and was generated using direct synthesis.

### 2.4 Immunohistochemistry, IHC

Immunohistochemistry was performed on 15 paraffin‐embedded human breast cancer and paracarcinoma tissue or breast cancer tissue microarrays purchased from Bioaitech (F261101; F151Br01, Xi'an, China). CHDH antibody was diluted 1:100 with primary antibody diluent. The secondary antibody was biotin‐labelled goat anti‐mouse/rabbit IgG. Immunohistochemistry staining was performed after determining the experimental conditions, following the protocol described previously [30]. The associated antibodies used were listed in Table S3.

### 2.5 Cell Culture

MDA‐MB‐231 cells were cultured with DMEM medium (Gibco, Thermo scientific, MA, USA) supplemented with 10% FBS (Gibco) and 1% P/S (Gibco). MDA‐MB‐453 and MDA‐MB‐468 cells were cultured with Leibovitz's L‐15 medium supplemented with 10% FBS (Gibco) and 1% P/S. The normal breast cell MCF‐10A was cultured with MCF‐10A Cell Complete Medium (Procell, Wuhan, China). All cell lines were grown at 37°C in the presence of 5% CO_2_. All cell lines were originally from ATCC and were mycoplasma‐free after testing.

### 2.6 Western Blot

Western blotting was performed according to a previously described protocol. The primary antibodies used are listed in Table S3.

### 2.7 Human Phospho‐Kinase Array

Relative kinase and protein phosphorylation levels were assessed using a Human Phosphokinase Array Kit (Cat. #ARY003C, R&D system, Minneapolis, MN, USA) following the manufacturer's instructions [30].

### 2.8 Wound Healing Assay

6‐well plate with 1 × 10^6^ cells per well and 2 mL cell suspension per well. After the cells had grown to about 90%–100%, the cells were scratched with a 10 μL tip compared to a straightedge, and after the scratches were made, the cells were washed twice with PBS and serum‐free basic medium was added. Photographs were taken under an inverted microscope as the width of the scratches at 0 h. The width of the scratches was analysed using Image J software after the same position was taken 24 h later to calculate the migration rate of the cells. Three independent experiments were performed.

### 2.9 Trans‐Well Assay

For migration assay, cells were collected by digestion, neutralisation and centrifugation, and 2 × 10^5^ bc cells containing 1% serum medium were added to Boyden chamber inserts of 8 μm pore membrane (Corning Incorporated, Corning, NY, USA). For invasion assay, Matrigel was added to the Boyden chamber insert with an 8 μm pore membrane, cells were collected by digestion, neutralisation and centrifugation, and 2 × 10^5^ bc cells containing 1% serum medium were added to the top of Matrigel. Culture medium containing 10% FBS was added to the lower chamber. After being placed in the incubator for 24 h, the chambers were removed for washing, fixation and staining with 0.1% crystal violet stain overnight and were washed three times with PBS. Cotton swabs were used to wipe off excess PBS in the chambers, and photographs were taken with an inverted microscope. Three independent experiments were performed.

### 2.10 Transplantation Tumour Model in Nude Mice

All in vivo mouse experiments were approved by the Animal Ethics Committee of Jining Medical University. Female BALB/c‐nu nude mice purchased at 6–8 weeks of age were randomly assigned to different groups (*n* = 6) and injected subcutaneously with 3 × 10^6^ cells per nude mouse. From day 15 (tumour palpable), mice tumours were measured (length and width with vernier callipers) every 2 days and data were recorded. The data were centrally counted and the volume of the tumours (mm^3^) was determined by the following formula: volume (mm^3^) = (width^2^ (mm^2^) × length (mm))/2. Nude mice were humanely euthanised using Avertin (Sigma‐Aldrich, Shanghai, China). For further analysis, tumours and lung tissues removed from nude mice were fixed in formalin, embedded in paraffin, and sectioned [31, 32].

### 2.11 Statistical Analysis

Data were analysed using GraphPad Prism9 software (GraphPad Software, San Diego, CA, USA). The values are expressed as mean ± standard error of mean (SEM). *p* values were calculated using *t*‐tests (two groups) and one‐way ANOVA (more than two groups) unless otherwise stated. *p* values below 0.05 were considered statistically significant. *p < 0.05, **p < 0.01, ***p < 0.001, ns: no statistical difference.

## 3 Results

### 3.1 CHDH Was Upregulated in Human Breast Cancer

**FIGURE 1:**
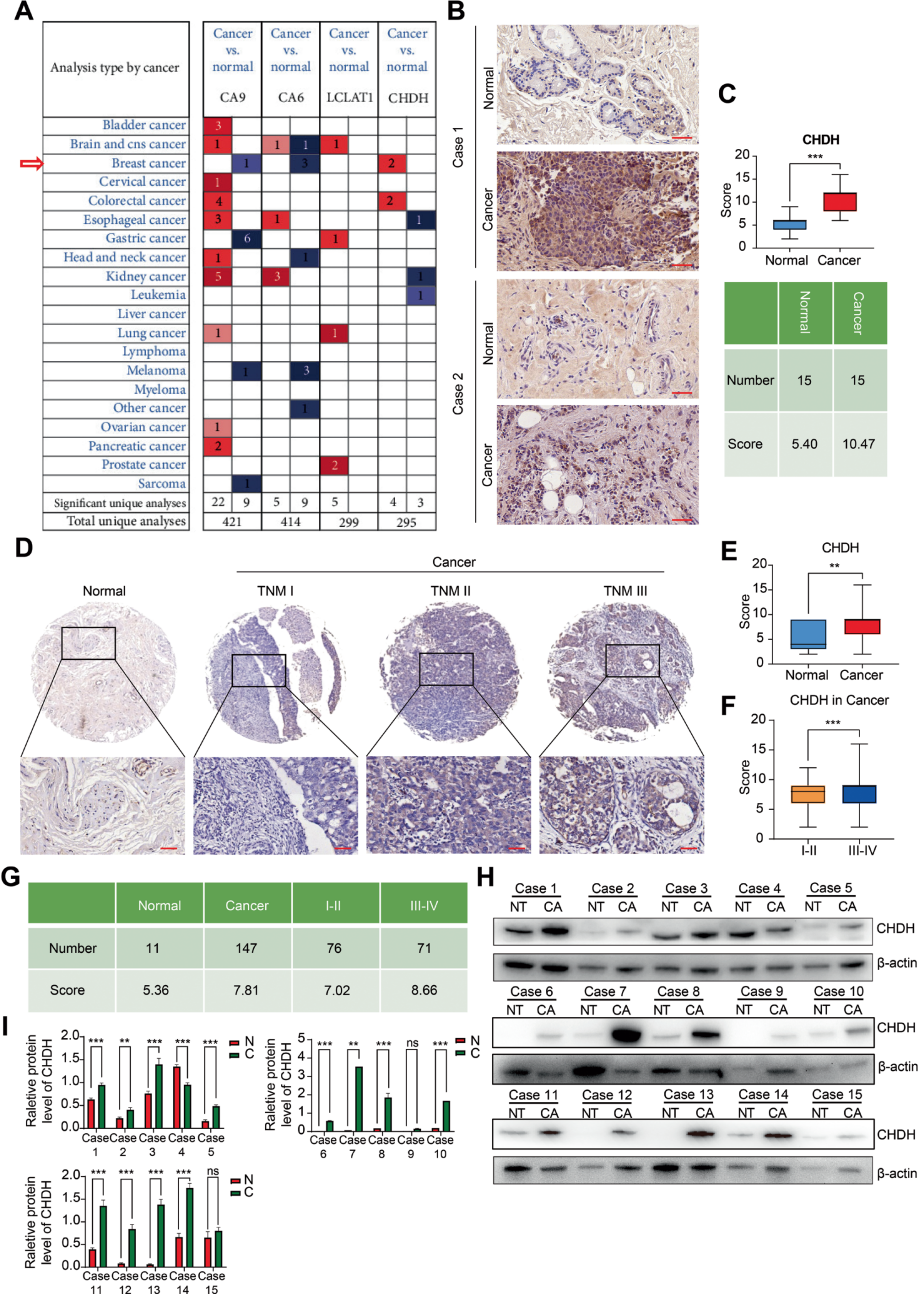
CHDH was highly expressed in human BC tissues. (A) ONCOMINE online databases were used to analyse the transcriptional expression of CHDH in breast cancers (cancer versus normal tissue). (B) Representative IHC images of CHDH in human fresh breast cancer tissue. Scale bar: 50 μm. (C) Quantification results of CHDH expression in fresh breast cancer tissue were shown. (D) Representative IHC images of CHDH in human BC tissue microarray. Scale bar: 50 μm. (E) Quantification results of CHDH expression in the BC tissue array were shown. (F, G) The correlation of CHDH expression and tumour TNMs was quantified. (H, I) Analysis of CHDH expression in 15 pairs of fresh breast cancer tissue and adjacent normal tissue by western blotting and statistical results.

To explore the expression pattern of CHDH in BC, we first conducted a search by bioinformatics analysis in the ONCOMINE online database. The results showed that CHDH expression in breast cancer tissues was significantly higher than in comparable normal tissues in the online database (Figure 1A). To validate the data obtained from public data, we performed immunohistochemical staining with CHDH‐specific antibodies on 15 pairs of breast cancer and adjacent tissue samples collected from clinical sources (Figure 1B,C), as well as on a human breast cancer tissue microarray containing 158 samples (11 normal tissues and 147 breast cancer tissues). The findings indicated that the expression level of the CHDH protein was significantly increased in breast cancer tissues in comparison to normal tissues (Figure 1D,E). Moreover, we analysed the association between TNM staging and CHDH protein expression in breast cancer. The results showed a significant positive correlation between TNM staging and elevated CHDH protein expression (Figure 1F,G). To further validate the above results, we examined CHDH expression by Western blot in 15 pairs of fresh breast cancer and adjacent non‐tumour tissues, and the findings were consistent with the above results, i.e., CHDH was highly expressed in the majority of breast cancer cells compared to non‐tumour tissues (Figure 1H,I). Together, these findings demonstrate that CHDH may be a potential factor promoting tumour function and is highly expressed in human BC tissues.

### 3.2 CHDH Deficiency Inhibited Tumour Cell Migration In Vitro

**FIGURE 2:**
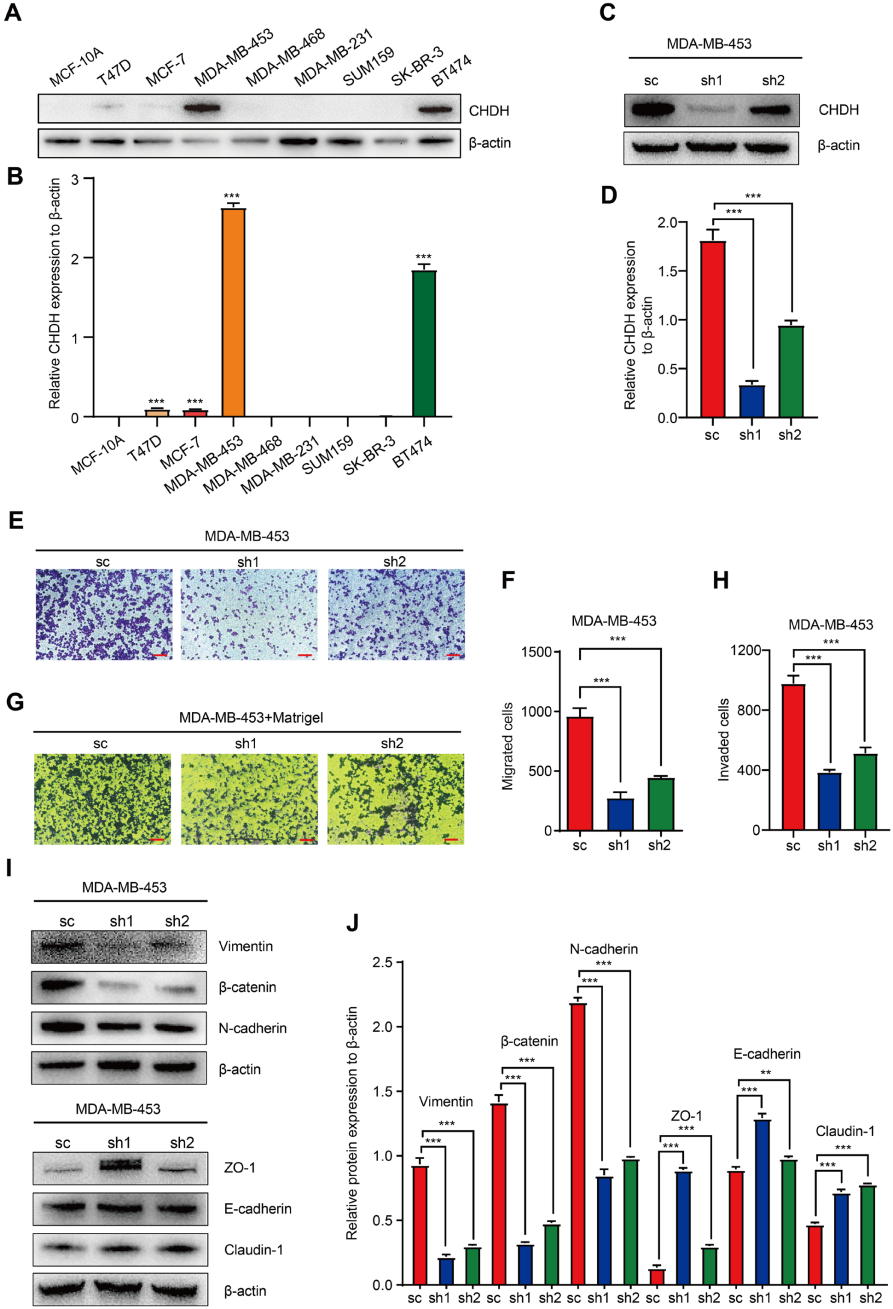
Silencing of CHDH inhibits BC cell migration in vitro. (A, B) Western blot analysis of CHDH expression in BC cell lines and normal breast cell lines; the quantification results were shown. (C, D) Western blot analysis of CHDH knockdown efficiency in MDA‐MB‐453 cell lines and statistical results were shown. (E, F) Representative images of trans‐well assay and statistical results on migrated cells MDA‐MB‐453‐shCHDH or shctrl were shown. Scale bar: 50 μm. (G, H) Representative images of invasion assay and statistical results on invaded cells in MDA‐MB‐453‐shCHDH or shctrl were shown. Scale bar: 50 μm. (I, J) Western blot analysis of the expression of EMT markers in MDA‐MB‐453 expressing shCHDH or shctrl cells; the quantification results were shown.

To verify whether the findings in tumour samples were consistent with BC cell lines. We selected normal breast epithelial cells MCF‐10A and several different breast cancer cell lines for culture; total cell protein extraction was performed separately. The expression of CHDH in normal mammary cells and breast cancer cell lines was detected by Western blot. The experimental results showed that CHDH protein was highly expressed in a variety of breast cancer cell lines compared to normal breast cells MCF‐10A (Figure 2A,B), especially in MDA‐MB‐453 cells. Therefore, this cell line could serve as a suitable model to determine the effect of CHDH on tumour migration in vitro.

To investigate the functional role of CHDH in BC tumour migration in vitro, we designed and constructed a CHDH‐specific shRNA and constructed a cell line with stable knockdown of CHDH in MDA‐MB‐453 by lentiviral system (Figure 2C,D). The results of the Trans‐well assay indicated that, in comparison to the control group, there was a significant decrease in the number of migrating MDA‐MB‐453 cells following the knockdown of CHDH (Figure 2E,F). Additionally, findings from the Matrigel‐mediated cell invasion assay demonstrated a notable reduction in the number of invading cells subsequent to CHDH knockdown (Figure 2G,H). Western blotting was performed to detect the expression of metastasis‐related epithelial‐mesenchymal transfer (EMT) markers. The results showed that the expression levels of epithelial cell‐associated molecular markers E‐cadherin, ZO‐1 and Claudin‐1 increased after the reduction of CHDH knockdown, while the expression levels of mesenchymal cell‐associated molecular markers N‐cadherin, β‐catenin and Vimentin decreased (Figure 2I,J). Taken together, our results suggest that CHDH plays an important role in regulating tumour migration in vitro.

### 3.3 Ectopic Expression of CHDH Promoted Tumour Migration In Vitro

**FIGURE 3:**
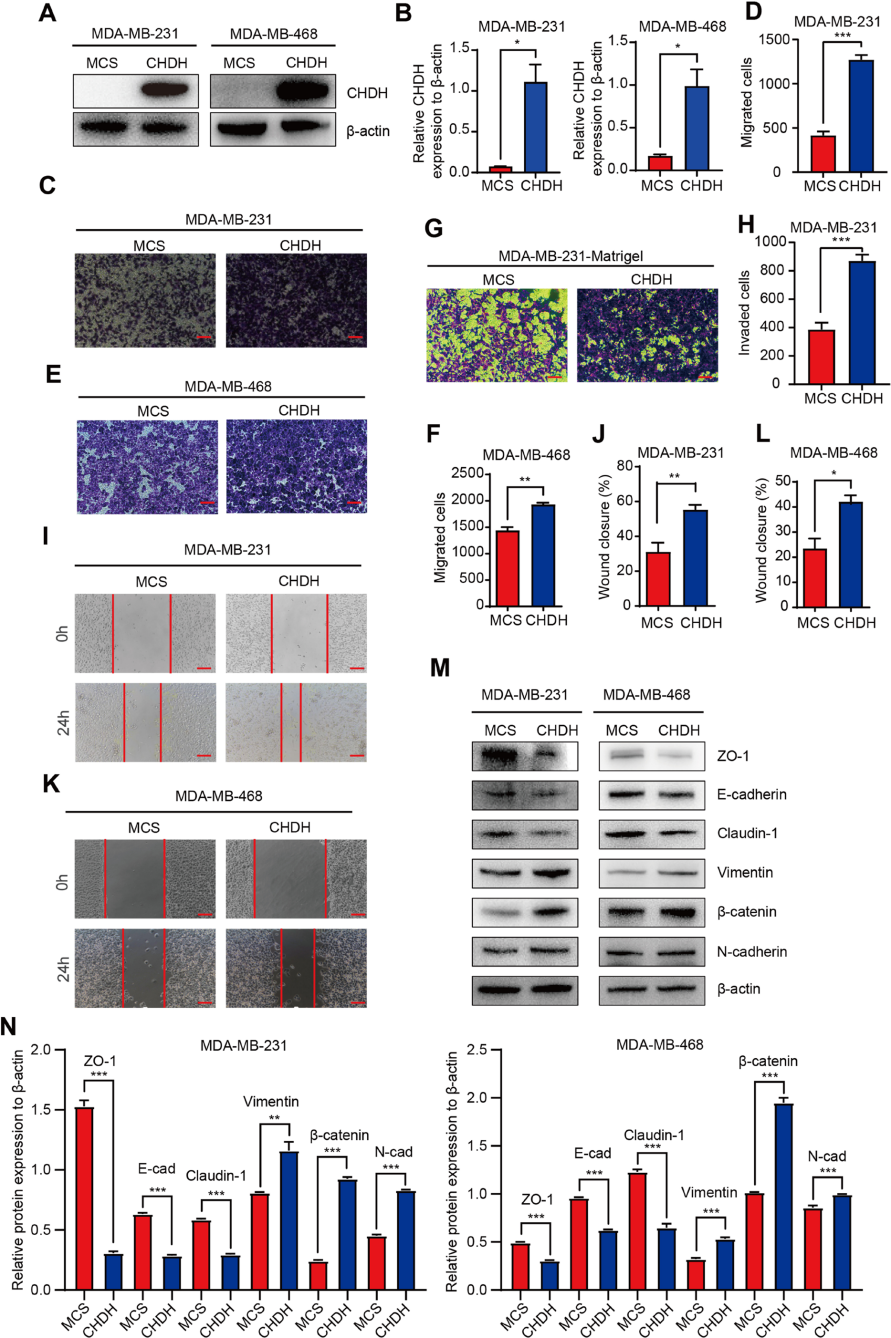
Ectopic expression of CHDH facilitates BC cell migration in vitro. (A, B) Western blot analysis of CHDH overexpression efficiency in MDA‐MB‐231 and MDA‐MB‐468 cell lines and statistical results. (C, D). Representative images of trans‐well assay and statistical results on migrated cells in MDA‐MB‐231 expressing CHDH or MCS were shown. Scale bar: 50 μm. (E, F) Representative images of trans‐well assay and statistical results on migrated cells in MDA‐MB‐468 expressing CHDH or MCS were shown. Scale bar: 50 μm. (G, H) Representative images of invasion assay and statistical results on invaded cells in MDA‐MB‐231 expressing CHDH or MCS were shown. Scale bar: 50 μm. (I, J) Representative images of wound healing assay and statistical results on migrated cells in MDA‐MB‐231 expressing CHDH or MCS were shown. Scale bar: 50 μm. (K, L) Representative images of wound healing assay and statistical results on migrated cells in MDA‐MB‐468 expressing CHDH or MCS were shown. Scale bar: 50 μm. (M, N) Western blot to analyse the expression of EMT markers in MDA‐MB‐231 and MDA‐MB‐468 expressing CHDH or MCS cells; the quantification results were shown.

To further validate the above results, we constructed overexpression CHDH cell lines by lentiviral infection in MDA‐MB‐231 and MDA‐MB‐468 cells (Figure 3A,B). The results by Trans‐well assay showed a statistically significant increase in the number of migrating MDA‐MB‐231 and MDA‐MB‐468 cells after overexpression of CHDH compared with the control group (Figure 3C–F). Furthermore, findings derived from Matrigel‐mediated cell invasion assays conducted on MDA‐MB‐231 cells indicated a substantial increase in the number of invasive cells following the overexpression of CHDH (Figure 3G,H). In addition, the wound healing assay demonstrated a higher and statistically significant wound closure in the overexpression group (Figure 3I–L). Compared with normal cells, the expression levels of EMT‐related markers showed that overexpression of CHDH increased the expression of mesenchymal cell‐related molecular markers N‐cadherin, β‐catenin and Vimentin and decreased the expression levels of epithelial cell‐related molecular markers E‐cadherin, ZO‐1 and Claudin‐1 (Figure 3M,N). The above results indicated that overexpression of CHDH promotes the migration of BC cells.

### 3.4 CHDH Facilitated Tumour Metastasis In Vivo

**FIGURE 4:**
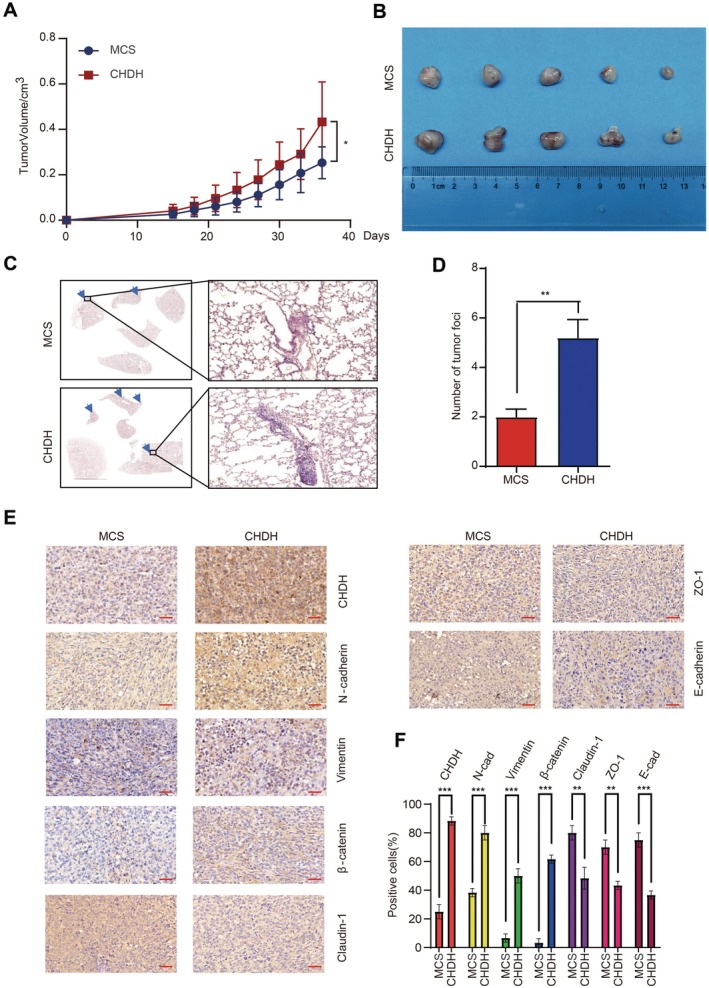
Ectopic expression of CHDH promotes BC metastasis in vivo. (A) Nude mice were injected with stable MDA‐MB‐231‐MCS and MDA‐MB‐231‐CHDH cells at the fourth fat pad; the tumour growth curves were measured. (B) The tumours separated from different group mice were shown. (C) Representative images of H&E staining on lungs from MDA‐MB‐231‐MCS and MDA‐MB‐231‐CHDH groups were shown. (D) Statistical results of metastatic nodules in the lungs from MDA‐MB‐231‐MCS and MDA‐MB‐231‐CHDH groups were shown. (E) IHC staining assay was conducted to check the expression of CHDH, ZO‐1, E‐cadherin, β‐catenin, N‐cadherin, Vimentin and Claudin‐1 in various group mice tumours. Scale bar: 50 μm. (F) The statistical results of CHDH, ZO‐1, E‐cadherin, β‐catenin, N‐cadherin, Vimentin and Claudin‐1 positive cells in various group mice tumours were shown.

As CHDH has been shown to promote BC cell migration in vitro, we investigated the effect of overexpression of CHDH on tumour growth and metastasis in vivo by using a nude mice breast cancer transplantation model. To this end, we injected MDA‐MB‐231‐MCS and MDA‐MB‐231‐CHDH stable cells into the fourth pair of fat pads on the right side of the nude mice respectively, and after the formation of the tumours, we recorded the growth process of the tumours and executed the nude mice on the 36th day. Compared with the MDA‐MB‐231‐MCS group, the MDA‐MB‐231‐CHDH group displayed faster tumour growth (Figure 4A), larger tumour volume (Figure 4B), and a higher number of tumour lung metastases (Figure 4C,D). The IHC results showed that overexpression of CHDH increased N‐cadherin, β‐catenin and Vimentin expression and decreased the expression of E‐cadherin, ZO‐1 and Claudin‐1, which was consistent with the in vitro findings (Figure 4E,F). Collectively, we conclude that overexpression of CHDH facilitated breast cancer metastasis in vivo.

### 3.5 Overexpression of CHDH Promotes CREB1 Activation

**FIGURE 5:**
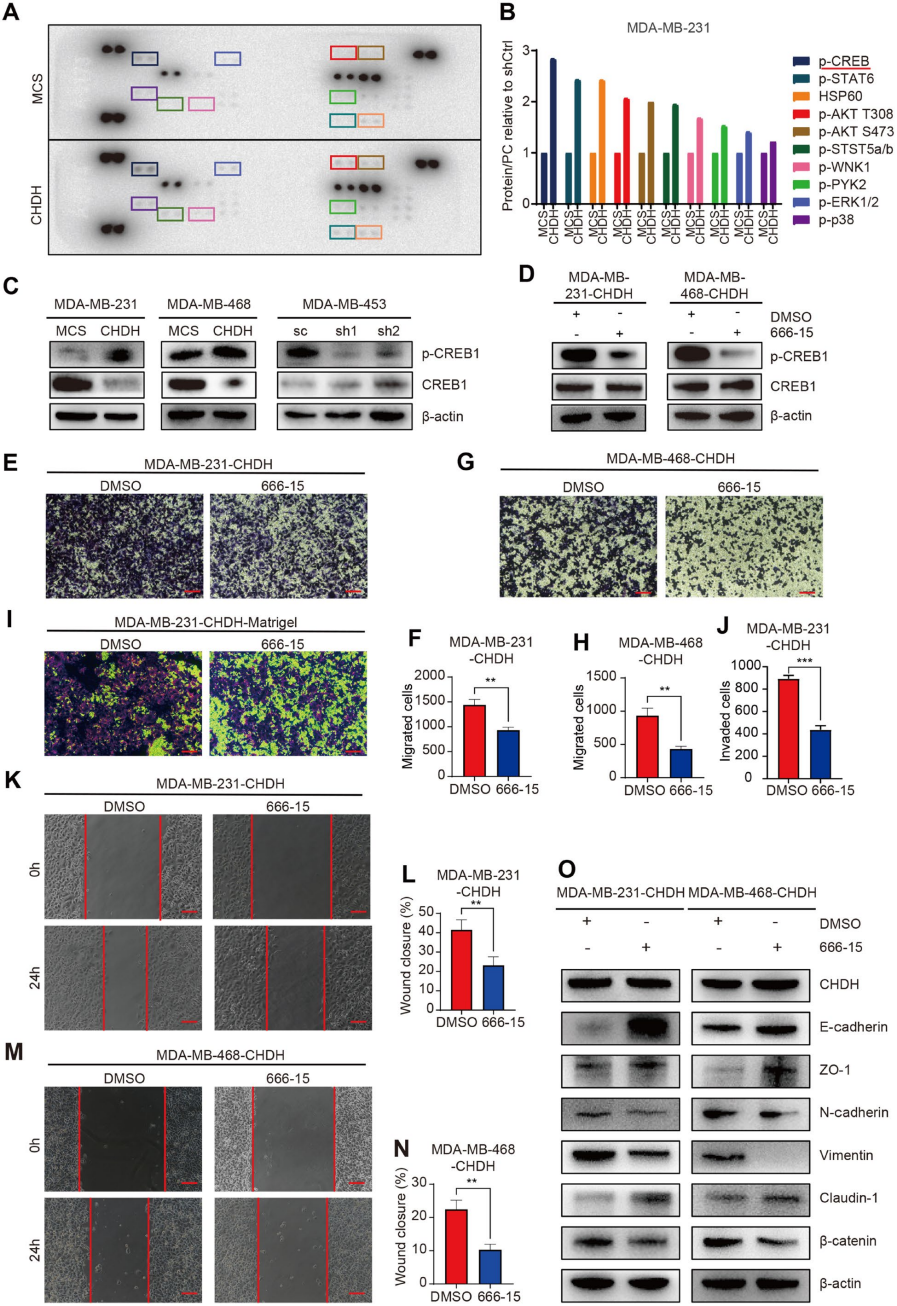
CHDH mediates CREB activation to promote BC cell migration in vitro. (A, B) Human phosphokinase array was performed to detect 39 kinases activation in MDA‐MB‐231 cells overexpressing CHDH or MCS. (C) Western blot analysis of the expression of p‐CREB1 and CREB in CHDH knockdown or overexpression cells. (D) Western blot analysis of the expression of p‐CREB1 and CREB in CHDH overexpression cells treated with 666‐15. (E, F) Representative images of the wound healing assay and statistical results on migrated cells in MDA‐MB‐231 expressing CHDH treated with 666‐15 were shown. Scale bar: 50 μm. (G, H) Representative images of the trans‐well assay and statistical results on migrated cells in MDA‐MB‐468 expressing CHDH treated with 666‐15 were shown. Scale bar: 50 μm. (I & J) Representative images of the invasion assay and statistical results on invaded cells in MDA‐MB‐231 expressing CHDH treated with 666‐15 were shown. Scale bar: 50 μm. (K, L) Representative images of the wound healing assay and statistical results on migrated cells in MDA‐MB‐231 expressing CHDH treated with 666‐15 were shown. Scale bar: 50 μm. (M, N) Representative images of the wound healing assay and statistical results on migrated cells in MDA‐MB‐468 expressing CHDH treated with 666‐15 were shown. Scale bar: 50 μm. (O) Western blot analysis of the expression of EMT markers in MDA‐MB‐231 and MDA‐MB‐468 expressing CHDH cells treated with 666–15.

To explore the potential molecular mechanisms of CHDH‐mediated BC progression, we performed a human phospho‐kinase array with MDA‐MB‐231‐MCS and MDA‐MB‐231‐CHDH proteins, which contained 2 key proteins and 37 phosphorylated kinases. The results showed (Figure 5A,B) that overexpression of CHDH promoted the activation of 10 kinases, with the most pronounced change in the expression of p‐CREB. To further validate that CHDH mediated the activation of CREB1 in BC cell lines, we examined the expression of p‐CREB1 in MDA‐MB‐231, MDA‐MB‐468 and MDA‐MB‐453 stable cell lines by Western blot. The results showed that overexpression of CHDH increased CREB1 activation in MDA‐MB‐231 and MDA‐MB‐468 cell lines. In contrast, knockdown of CHDH decreased p‐CREB1 expression in the MDA‐MB‐453 cell line (Figure 5C, Figure S1A). These findings suggest that overexpression of CHDH promotes CREB1 activation in vitro.

To further validate the effect of CREB1 activation in CHDH‐mediated cell migration promotion, MDA‐MB‐231 and MDA‐MB‐468 overexpressing CHDH cells were treated with 1 μM of the CREB inhibitor 666‐15 for 12 h. The inhibitor efficiency was detected by Western blot and showed that CREB1 activation was inhibited after treatment with the specific inhibitor 666‐15 (Figure 5D, Figure S1B). Migration of MDA‐MB‐231‐CHDH and MDA‐MB‐468‐CHDH cells was statistically reduced after inhibition of CREB activation compared to the control, as shown by the trans‐well assay (Figure 5E–H). Moreover, findings derived from Matrigel‐mediated cell invasion assays conducted on MDA‐MB‐231‐CHDH cells indicated a substantial decrease in the number of invasive cells following the treatment of 666‐15 (Figure 5I,J). In addition, the results of the wound healing assay showed that the wound closure was decreased in the 666‐15 group compared to the control (Figure 5K–N). Western blot results showed that 666‐15 treatment rescued the expression of CHDH‐mediated EMT markers respectively (E‐cadherin, ZO‐1 and Claudin‐1 increased, N‐cadherin, β‐catenin and Vimentin decreased) (Figure 5O, Figure S1C). The above results indicated that CREB activation was responsible for CHDH‐mediated migration of BC cells.

### 3.6 CHDH Mediated Histone Methylation to Facilitate IL17RB/CREB Signalling Further to Promote BC Cell Migration In Vitro

**FIGURE 6:**
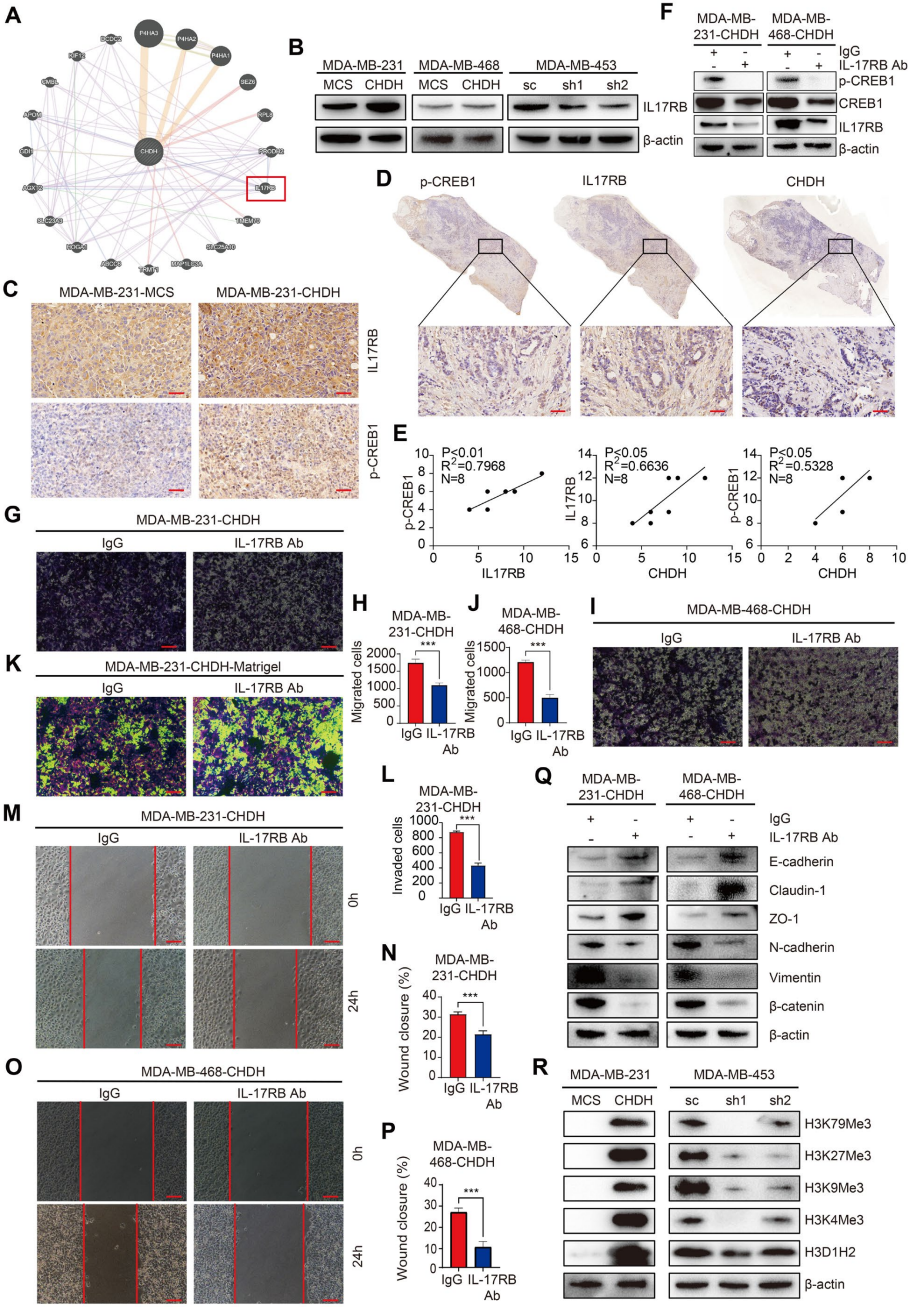
IL17RB was indispensable for CHDH‐mediated CREB activation and BC cell migration. (A) GENEMANIA online database was used to analyse CHDH co‐expression genes. (B) Western blot analysis of the expression of IL17RB in CHDH knockdown or overexpression cells. (C) Representative IHC staining results of IL17RB and p‐CREB1 in various groups of mice tumours, scale bar: 50 μm. (D, E) Representative IHC staining of p‐CREB1, IL17RB and CHDH in human BC tissues; the correlation results were shown, scale bar: 50 μm. (F) Western blot analysis of the expression of IL17RB, p‐CREB1, and CREB in CHDH overexpression cells treated with IL17RB Ab. (G, H). Representative images of trans‐well assay and statistical results on migrated cells in MDA‐MB‐231 expressing CHDH treated with IL17RB Ab were shown. Scale bar: 50 μm. (I, J). Representative images of trans‐well assay and statistical results on migrated cells in MDA‐MB‐468 expressing CHDH treated with IL17RB Ab were shown. Scale bar: 50 μm. (K, L) Representative images of invasion assay and statistical results on invaded cells in MDA‐MB‐231 expressing CHDH treated with IL17RB Ab were shown. Scale bar: 50 μm. (M, N) Representative images of wound healing assay and statistical results on migrated cells in MDA‐MB‐231 expressing CHDH treated with IL17RB Ab were shown. Scale bar: 50 μm. (O, P) Representative images of wound healing assay and statistical results on migrated cells in MDA‐MB‐468 expressing CHDH treated with IL17RB Ab were shown. Scale bar: 50 μm. (Q) Western blot analysis of the expression of EMT markers in CHDH overexpression cells treated with IL17RB Ab. (R) Western blot analysis of the expression of tri‐methylation of histone H3 in CHDH knockdown or overexpression cells.

To investigate the mechanism of CREB activation by CHDH, we analysed the set of CHDH‐related genes using the GeneMANIA online database. Among the resulting 20 genes associated with CHDH function, IL17RB may be associated with CREB activation (Figure 6A). It has been previously demonstrated that IL17 binding to IL17R may activate CREB and thus promote cell migration [29]. We verified the expression of IL17RB in BC cell lines overexpressing and knocking down CHDH by Western blot. The results showed that IL17RB expression was increased in breast cancer cell lines overexpressing CHDH. Knockdown of CHDH significantly decreased IL17RB expression (Figure 6B, Figure S2A). Moreover, the IHC results showed higher levels of IL17RB and CREB1 expression in tumours of mice overexpressing CHDH (Figure 6C). To confirm the correlation between p‐CREB, IL17RB and CHDH, IHC staining was performed on 8 human BC samples to detect the co‐expression of p‐CREB1, IL17RB and CHDH. The results showed that CHDH with p‐CREB1, CHDH with IL17RB, p‐CREB1 with IL17RB were positively correlated in BC samples (Figure 6D,E).

To evaluate the role of IL17RB expression in CREB activation and CHDH‐mediated BC cell migration, IL17RB expression was blocked using an IL17RB‐specific antibody. Western blot results showed that the IL17RB antibody effectively inhibited IL17RB expression and CREB1 activation in MDA‐MB‐231‐CHDH and MDA‐MB‐468‐CHDH cells (Figure 6F, Figure S2B). Migration of MDA‐MB‐231‐CHDH and MDA‐MB‐468‐CHDH cells was statistically reduced after the treatment of IL17RB antibody compared to the control, as shown by trans‐well assay (Figure 6G–J). Moreover, findings derived from Matrigel‐mediated cell invasion assays conducted on MDA‐MB‐231‐CHDH cells indicated a substantial decrease in the number of invasive cells following the treatment of IL17RB antibody (Figure 6K,L). In addition, the results of the wound healing assay showed that the wound closure was decreased in the IL17RB group compared to the control (Figure 6M–P). Western blot results showed that IL17RB antibody blockade significantly rescued CHDH‐mediated expression of EMT markers respectively (E‐cadherin, ZO‐1 and Claudin‐1 increased, N‐cadherin, β‐catenin and Vimentin decreased) (Figure 6Q, Figure S2C). This effect may further regulate the expression of downstream target genes. Previous studies have shown that CHDH is one of the key enzymes in choline metabolism to produce methyl donors [3, 4]. Histone H3 methylation is a post‐translational modification of chromatin that plays a key role in the regulation of gene expression. We examined the effect of CHDH expression on histone H3 trimethylation using western blotting and methylation antibody kits. The results showed that ectopic expression of CHDH affected the trimethylation of histone H3 at K4, K9, K27 and K79 compared with control (Figure 6R, Figure S2D). Collectively, these results revealed that CHDH mediated histone methylation to facilitate IL17RB/CREB signalling further to promote BC cell migration in vitro.

### 3.7 Proposed Model of CHDH Promotes BC Metastasis

**FIGURE 7:**
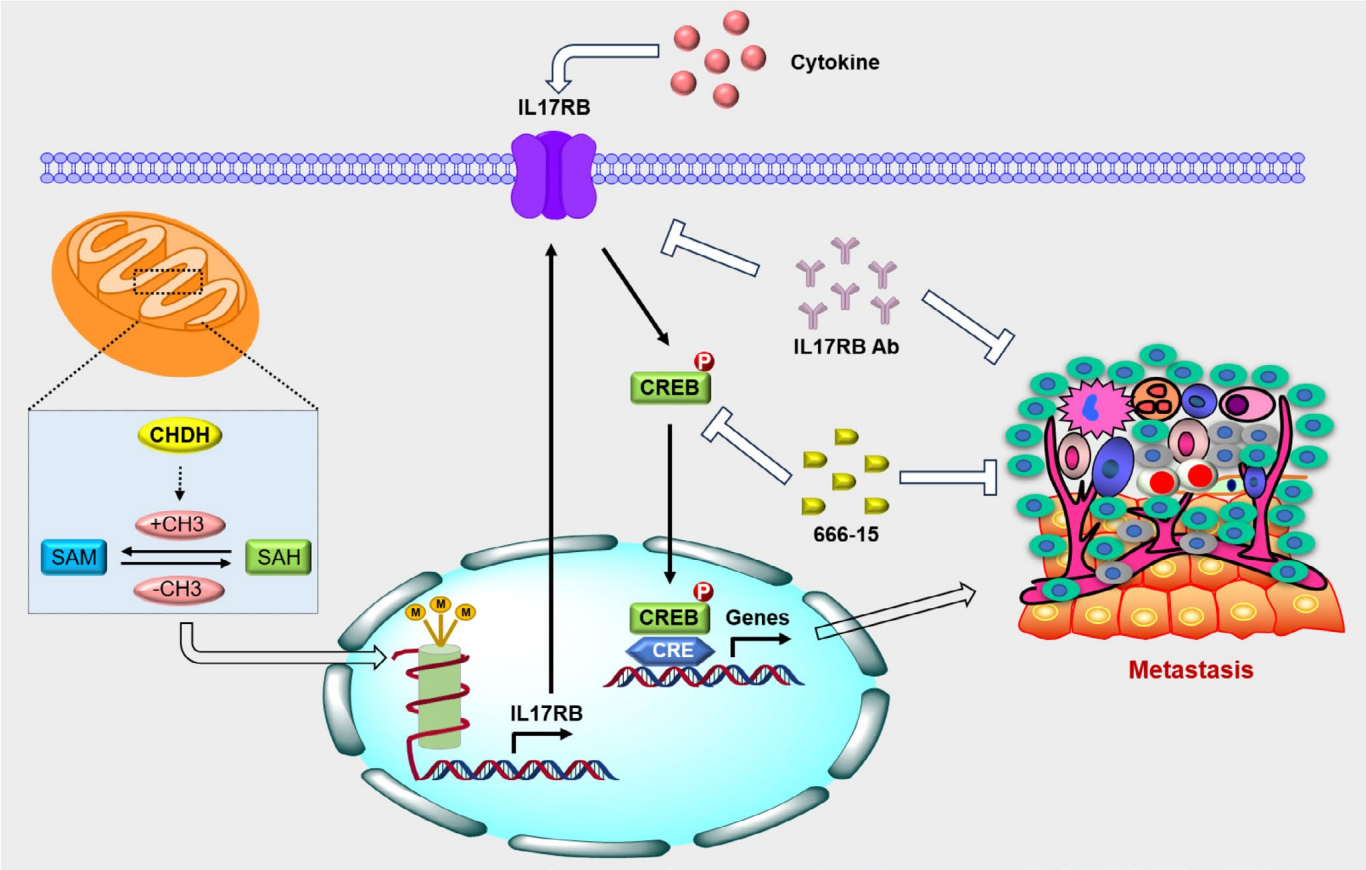
Proposed model of CHDH in mediating BC metastasis.

We propose the following model based on the findings in our study (Figure 7): CHDH is one of the major enzymes of the methyl donor pathway, may affect the trimethylation of histone H3, which in turn regulates the expression of the downstream target gene IL17RB. IL17RB promotes downstream CREB activation and promotes BC metastasis. As a therapy strategy, IL17RB antibody targets IL17RB and 666‐15 targets CREB to abolish CHDH‐mediated BC metastasis and progression.

## 4 Discussion

Breast cancer ranks among the most prevalent malignant tumours affecting women [1]. As research on breast cancer advances, a variety of diagnostic targets [33, 34, 35] and therapeutic strategies [36, 37, 38] have been identified; nevertheless, metastasis continues to be a primary contributor to mortality in patients with breast cancer. The identification of novel early diagnostic targets, along with a comprehensive understanding of their molecular mechanisms and the development of targeted therapeutic approaches, is essential for establishing a theoretical framework and effective interventions for metastatic breast cancer. Consequently, this study seeks to investigate the potential of the CHDH molecule as a novel biomarker for breast cancer metastasis.

CHDH, as one of the key enzymes involved in choline metabolism in mitochondria, affects human growth and development by participating in choline metabolism and mitochondrial autophagy. The biochemical characterisation of CHDH has been poorly studied due to the lack of a stable, active and highly purified enzyme [7]. Wu et al. found that the high CHDH expression was correlated with the prognosis of patients with head and neck cell carcinoma [39]. Ma et al. [40] compared the genes of patients with early stage Oestrogen Receptor (ER)‐positive breast cancer who did not have recurrence of breast cancer after tamoxifen treatment with those of patients with recurrent breast cancer, and found that there was a significant difference in the expression of CHDH. In a study of novel prognostic biomarkers for breast cancer, CHDH expression was found to be regulated by oestrogen [41]. CHDH as a prognostic biomarker for evaluating breast cancer may be associated with ER^+^ breast cancer. However, the expression level and mechanism of action of CHDH in BC tissues have not been reported, and its relationship with BC metastasis is unclear. Our results demonstrated that CHDH was highly expressed in BC tissues and positively correlated with BC TNM stage. Moreover, CHDH promotes BC progression and metastasis as a potential proto‐oncogene.

CREB has been extensively studied in neurological injury [42], memory [43] and malignant tumours [44]. A study showed that CREB1 levels were significantly elevated in tumour tissues of breast cancer patients and were strongly associated with poor prognosis, breast cancer metastasis and lymph node involvement [45]. CREB1 also functions as a transcription factor to regulate a variety of miRNAs. Zhang et al. [44] first suggested that CREB1 is a downstream effector of miR‐450a mediating growth and metastatic traits in breast cancer cells, and that elevated CREB1 expression is associated with poor ER+ breast cancer prognosis. Moreover, CREB1 was found to play an important role in neurons that can be activated by acetylcholine‐like neurotransmitters [46]. Importantly, CHDH expression in breast cancer is also regulated by oestrogen [41]; both CHDH and acetylcholine are involved in choline metabolism, suggesting a potential association between CREB1 and CHDH. However, the role of CREB1 in CHDH‐mediated BC progression has not been reported. We found that p‐CREB1 was highly expressed in BC tissues overexpressing CHDH in nude mice by IHC staining. As a potentially relevant gene for CHDH, the expression of p‐CREB1 was altered with CHDH expression. Our results demonstrated that CHDH may mediate BC metastasis by activating CREB. This may provide new concepts and evidence for the development of relevant therapeutic approaches against CHDH.

Overexpression of membrane receptors is a common characteristic in different cancer types, such as ER and IL17RB in breast cancer [47]. MA et al. [40] found that the total isoforms of IL17RB, including membrane‐bound and secreted IL17RB isoform 1, promote breast carcinogenesis. Moreover, both IL17RB and CHDH are oestrogen‐regulated genes [48]. The IL17RB gene locus is identical to the coding gene position of CHDH (gene position 3p21.1) [3, 49]. CHDH may exert its pro‐carcinogenic effects through IL17RB [31]. Our study found that the expression of IL17RB was also significantly increased after overexpression of CHDH. Some recent studies have found that IL17 binding to the receptor is promoted after external stimulation, facilitating the activation of CREB [50, 51]. Our findings suggest a high correlation between CREB1 activation and IL17RB expression. We hypothesised that CHDH activates CREB1 by promoting the expression of IL17RB, which may reveal a novel mechanism by which CHDH promotes tumour progression. Nevertheless, as previously reported for breast cancer with highly suspicious microcalcifications on mammography [52, 53], the mechanism regulating stage‐specific expression levels of CHDH/CHDH‐induced IL17RB and activation (phosphorylation) status of CREB1 should be further explored to provide a theoretical basis for CHDH as a diagnostic and therapeutic target for metastatic breast cancer.

CHDH, an essential enzyme integral to mitochondrial metabolism, is situated within the inner mitochondrial membrane. It exerts its influence by modulating molecular expression and activating downstream signalling pathways, a process characterised by its complexity. In the present study, we propose that CHDH modifies histone methylation levels by affecting the equilibrium of methyl donors and acceptors, thereby regulating the expression of downstream target genes, such as IL17RB. It is important to note that this process may not be entirely precise and could necessitate the application of histone methyltransferase inhibitors to substantiate the findings. To further elucidate the mechanisms underlying CHDH's function, future research will employ RNA sequencing analysis to identify pertinent downstream target genes and associated signalling pathways.

## 5 Conclusion

In conclusion, we proposed a novel finding that CHDH, as one of the major enzymes of the methyl donor pathway, affects histone H3 trimethylation, which in turn regulates the expression of the downstream target gene IL17RB. IL17RB promotes downstream CREB activation and promotes BC metastasis. As a therapy strategy, IL17RB antibody targets IL17RB and 666‐15 targets CREB to abolish CHDH‐mediated BC metastasis and progression. Our findings indicate that CHDH could serve as a novel therapeutic target in the treatment of metastatic BC.

## Author Contributions

**Yifei Li:** data curation (equal), formal analysis (equal), writing – original draft (equal). **Yipeng Liu:** data curation (equal). **Xiaowen Yang:** data curation (equal), project administration (equal). **Xinzhuang Shen:** formal analysis (equal). **Zongjun Liu:** data curation (equal). **Yuqiu Ma:** resources (equal). **Yiting Zhan:** data curation (equal), formal analysis (equal). **Xinyu Jiang:** methodology (equal), resources (equal). **Chengjin Liang:** data curation (equal). **Xiaoyuan Zhang:** funding acquisition (equal), writing – review and editing (equal). **Huan Liu:** writing – review and editing (equal). **Wenzhi Shen:** data curation (equal), funding acquisition (equal), writing – review and editing (equal).

## Ethics Statement

The tumour sample tissue studies were approved by the Institutional Ethics Committee of Jining Medical University (number: JNMC‐2021‐YX‐017). The Animal Studies were conducted according to established animal welfare guidelines and approved by the Institutional Animal Committee of Jining Medical University (Number: JNMC‐2021‐DW‐039).

## Consent

The authors have nothing to report.

## Conflicts of Interest

The authors declare no conflicts of interest.

## Supporting information


**Figure S1:** The quantification results of western blot in Figure 5.
**Figure S2:** The quantification results of western blot in Figure 6.
**Table S1:** Patients information.
**Table S2:** Primer sequences.
**Table S3:** Antibodies list.

## Data Availability

The data that support the findings of this study are available from the corresponding author upon reasonable request.
