# Supplementary material for: CHDH Promotes Breast Cancer Metastasis Relying on IL17RB/CREB1 Signalling Activation
Source: J Cell Mol Med. 2025 Sep 10;29(17):e70792. doi: 10.1111/jcmm.70792 (PMC12421421; doi:10.1111/jcmm.70792)
Supplement: Supplementary file 1 — Figure S1: The quantification results of western blot in Figure 5. Figure S2: The quantification results of western blot in Figure 6. Table S1: Patients information. Table S2: Primer sequences. Table S3: Antibodies list. [file JCMM-29-e70792-s001.docx]

***Supplementary Figures & Tables***

**CHDH promotes breast cancer metastasis relying on IL17RB/CREB1 signaling activation**

**
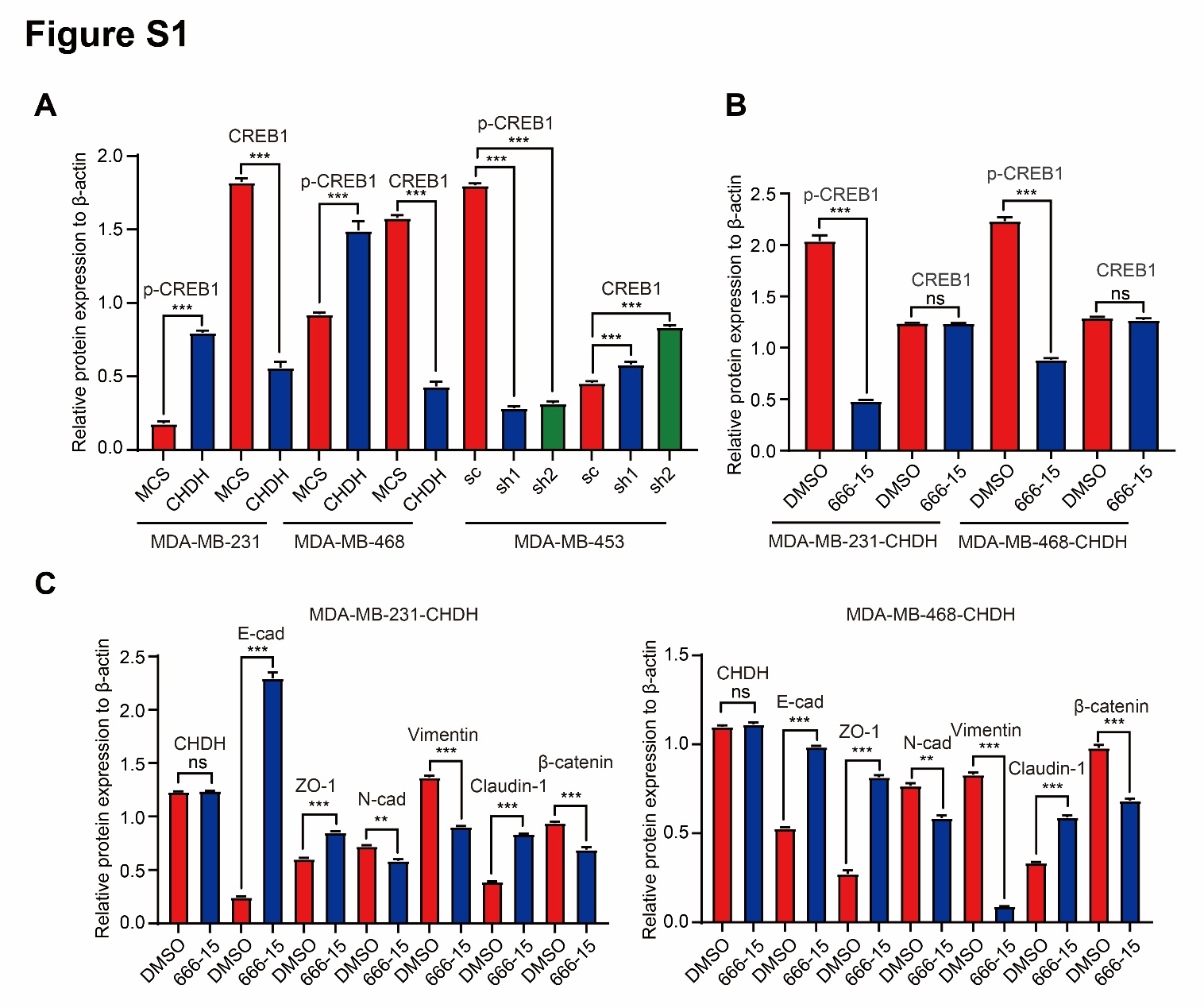
**

**Figure S1.** **The quantification results of western blot in figure 5.**

**A**. The quantification results of figure 5C were shown. **B**. The quantification results of figure 5D were shown. **C**. The quantification results of figure 5M were shown.

**
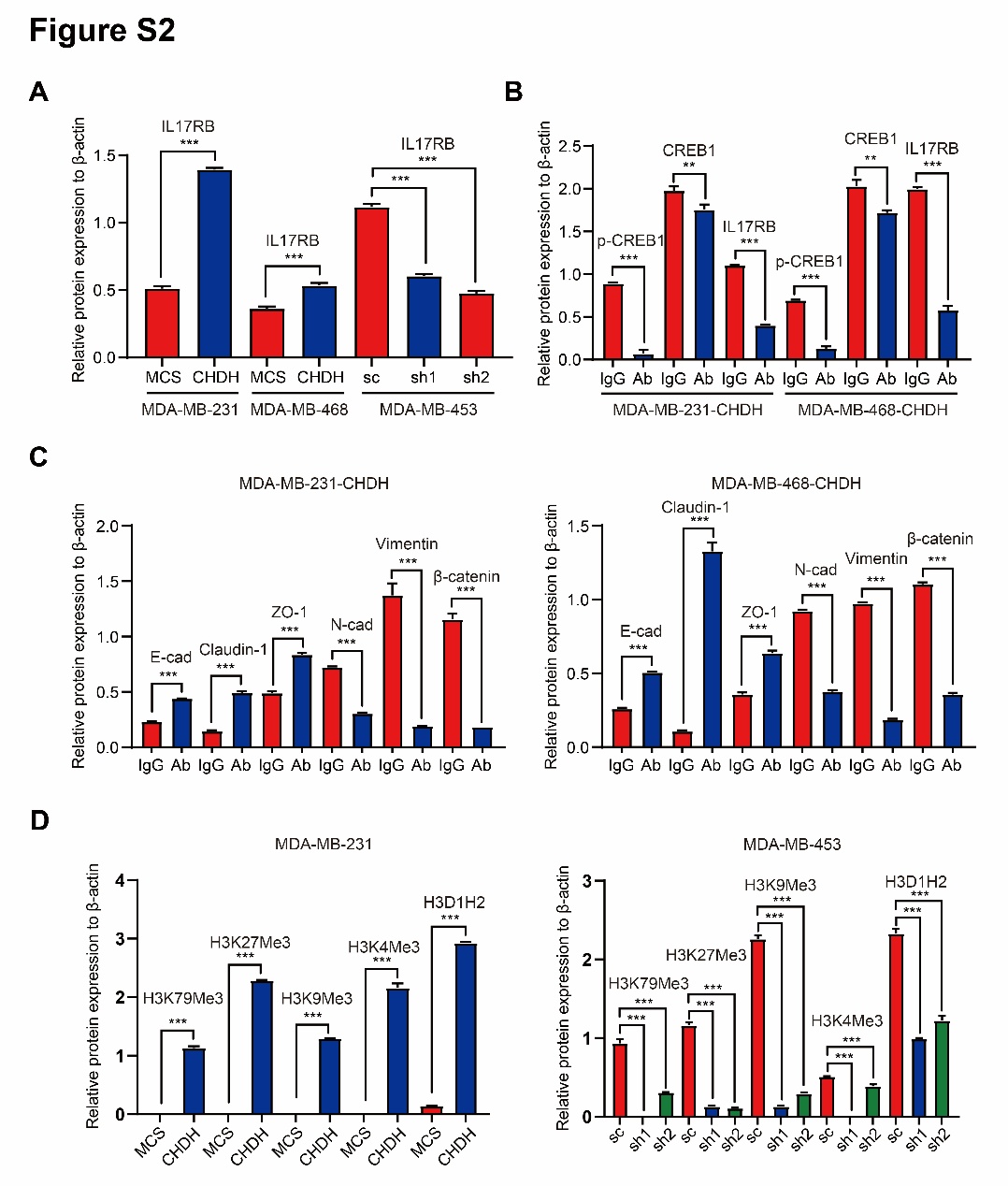
**

**Figure S2. The quantification results of western blot in figure 6.**

**A**. The quantification results of figure 6B were shown. **B**. The quantification results of figure 6F were shown. **C**. The quantification results of figure 6O were shown. **D**. The quantification results of figure 6P were shown.

**Table S1. Patients information**

| Sample Number | ID Number | Sex | Organ | Pathology Diagnosis | Type | Date |
| --- | --- | --- | --- | --- | --- | --- |
| #1 | 7101458720 | F | Breast | invasive breast carcinoma | Malignant | 7^th^ Mar, 2022 |
| #2 | 7101444795 | F | Breast | invasive breast carcinoma | Malignant | 8^th^ Mar, 2022 |
| #3 | 7101402305 | F | Breast | invasive breast carcinoma | Malignant | 8^th^ Mar, 2022 |
| #4 | 7101461815 | F | Breast | invasive breast carcinoma | Malignant | 9^th^ Mar, 2022 |
| #5 | 7101473372 | F | Breast | invasive breast carcinoma | Malignant | 10^th^ Mar, 2022 |
| #6 | 7101659245 | F | Breast | invasive breast carcinoma | Malignant | 9^th^ May, 2022 |
| #7 | 1001271308 | F | Breast | invasive breast carcinoma | Malignant | 11^th^ May, 2022 |
| #8 | 7101669482 | F | Breast | invasive breast carcinoma | Malignant | 11^th^ May, 2022 |
| #9 | 7101685888 | F | Breast | invasive breast carcinoma | Malignant | 13^th^ May, 2022 |
| #10 | 7101679860 | F | Breast | invasive breast carcinoma | Malignant | 13^th^ May, 2022 |
| #11 | 7101685650 | F | Breast | invasive breast carcinoma | Malignant | 19^th^ May, 2022 |
| #12 | 7100289628 | F | Breast | invasive breast carcinoma | Malignant | 7^th^ Apr, 2023 |
| #13 | 7102120317 | F | Breast | invasive breast carcinoma | Malignant | 7^th^ Apr, 2023 |
| #14 | 3001380506 | F | Breast | invasive breast carcinoma | Malignant | 26^th^ Apr, 2023 |
| #15 | 7102153046 | F | Breast | invasive breast carcinoma | Malignant | 28^th^ Apr, 2023 |

**Table S2. Primer sequences**

| Name | Sequence |
| --- | --- |
| CHDH-shRNA-1 | AAAAGGACATGACCATCCATGAAGGTTGGATCCAACCTTCATGGATGGTCATGTCC |
| CHDH-shRNA-2 | AAAAGCTTGTGAGCAGGGTGCTATTTTGGATCCAAAATAGCACCCTGCTCACAAGC |
| shCtrl | AAAAGCTACACTATCGAGCAATTTTGGATCCAAAATTGCTCGATAGTGTAGC |

**Table S3. Antibodies List**

| **Antibody** | | **Clone, Cat #** | **Vendor** | **City, State, Country** |
| --- | --- | --- | --- | --- |
| CHDH | Mouse monoclonal | sc-393885 | Santa Cruz Biotechnology | Santa Cruz, CA, USA |
| β-actin | Mouse monoclonal | sc-47778 | Santa Cruz Biotechnology | Santa Cruz, CA, USA |
| Epithelial-Mesenchymal Transition (EMT) Antibody Sampler Kit | Rabbit monoclonal | 9782 | Cell Signal Technology | Danvers, MA, USA |
| p-CREB1 | Rabbit polyclonal | 28792-1-AP | Proteintech | Wuhan, China |
| IL17RB | Rabbit polyclonal | 20673-1-AP | Proteintech | Wuhan, China |
| Tri-Methyl Histone H3Ab Sampler Kit | Rabbit monoclonal | 9783 | Cell Signal Technology | Danvers, MA, USA |
| CREB1 | Rabbit polyclonal | 12208-1-AP | Proteintech | Wuhan, China |
